# 2-(5-Methyl-3-methyl­sulfinyl-1-benzofuran-2-yl)acetic acid

**DOI:** 10.1107/S1600536809033765

**Published:** 2009-08-29

**Authors:** Hong Dae Choi, Pil Ja Seo, Byeng Wha Son, Uk Lee

**Affiliations:** a Department of Chemistry, Dongeui University, San 24 Kaya-dong Busanjin-gu, Busan 614-714, Republic of Korea; b Department of Chemistry, Pukyong National University, 599-1 Daeyeon 3-dong, Nam-gu, Busan 608-737, Republic of Korea

## Abstract

In the title compound, C_12_H_12_O_4_S, the O atom and the methyl group of the methyl­sulfinyl substituent are located on opposite sides of the plane of the benzofuran fragment. In the crystal structure, inter­molecular C—H⋯O and O—H⋯O hydrogen-bonding inter­actions are found. The structure also exhibits aromatic π–π inter­actions between the furan and benzene rings [centroid–centroid distance = 3.841 (5) Å].

## Related literature

For the crystal structures of similar alkyl 2-(5-methyl-3-methyl­sulfinyl-1-benzofuran-2-yl)acetate derivatives, see: Choi *et al.* (2008**a* ▶,b* ▶). For the pharmacological properties of benzofuran compounds, see: Howlett *et al.* (1999 ▶); Twyman & Allsop (1999 ▶). For natural products that contain benzofuran ring systems, see: Akgul & Anil (2003 ▶); von Reuss & König (2004 ▶).
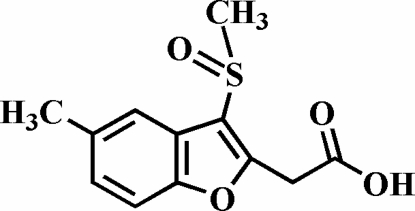


## Experimental

### 

#### Crystal data

C_12_H_12_O_4_S
*M* *_r_* = 252.28
Orthorhombic, 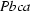

*a* = 7.767 (1) Å
*b* = 16.248 (2) Å
*c* = 18.733 (2) Å
*V* = 2364.1 (5) Å^3^
*Z* = 8
Mo *K*α radiation
μ = 0.27 mm^−1^
*T* = 293 K
0.40 × 0.20 × 0.05 mm

#### Data collection

Bruker SMART CCD diffractometer
Absorption correction: multi-scan (*SADABS*; Sheldrick, 2000 ▶) *T*_min_ = 0.899, *T*_max_ = 0.987
13669 measured reflections
2690 independent reflections
1461 reflections with *I* > 2σ(*I*)
*R*_int_ = 0.110

#### Refinement

*R*[*F* ^2^ > 2σ(*F* ^2^)] = 0.052
*wR*(*F* ^2^) = 0.156
*S* = 1.04
2690 reflections
160 parameters
H atoms treated by a mixture of independent and constrained refinement
Δρ_max_ = 0.35 e Å^−3^
Δρ_min_ = −0.45 e Å^−3^

### 

Data collection: *SMART* (Bruker, 2001 ▶); cell refinement: *SAINT* (Bruker, 2001 ▶); data reduction: *SAINT*; program(s) used to solve structure: *SHELXS97* (Sheldrick, 2008 ▶); program(s) used to refine structure: *SHELXL97* (Sheldrick, 2008 ▶); molecular graphics: *ORTEP-3* (Farrugia, 1997 ▶) and *DIAMOND* (Brandenburg, 1998 ▶); software used to prepare material for publication: *SHELXL97*.

## Supplementary Material

Crystal structure: contains datablocks global, I. DOI: 10.1107/S1600536809033765/nc2155sup1.cif

Structure factors: contains datablocks I. DOI: 10.1107/S1600536809033765/nc2155Isup2.hkl

Additional supplementary materials:  crystallographic information; 3D view; checkCIF report

## Figures and Tables

**Table 1:** Hydrogen-bond geometry (Å, °)

*D*—H⋯*A*	*D*—H	H⋯*A*	*D*⋯*A*	*D*—H⋯*A*
C9—H9*B*⋯O4^i^	0.97	2.48	3.339 (5)	148
O2—H2⋯O4^ii^	0.85 (6)	1.74 (6)	2.590 (4)	175 (5)

Symmetry codes: (i) 
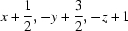
; (ii) 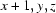.

**Fig. 1.**
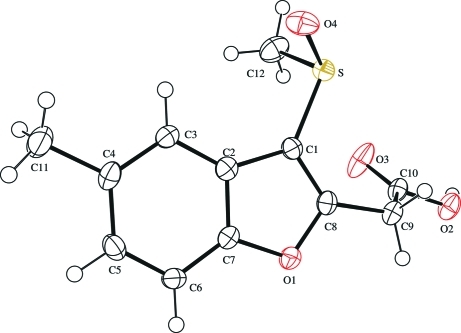
The molecular structure of the title compound with the atom numbering scheme. Displacement ellipsoids are drawn at the 50% probability level. H atoms are presented as a small cycles of arbitrary radius.

**Fig. 2.**
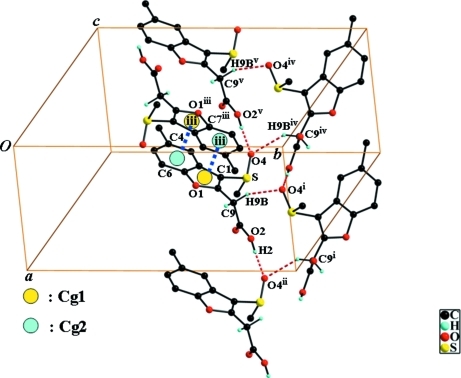
C–H···O, O–H···O, and π···π interactions (dotted lines) in the structure of the title compound. Cg denotes the ring centroid. [Symmetry codes: (i) - x + 2, - y + 1, - z + 1; (ii) x + 1/2, - y + 3/2, - z + 1; (iii) x + 1, y, z; (iv) x - 1/2, - y + 3/2, - z + 1; (v) x - 1, y, z.]

## supplementary crystallographic information

### Comment

Molecules containing benzofuran skeletons have been received considerable attention in the field of their pharmacological properties (Howlett et al.,  1999; Twyman & Allsop, 1999) and often occurs as natural products (Akgul & Anil, 2003; von Reuss & König, 2004). As part of our ongoing studies on the synthesis and structure of such compounds the structure of the title compound is reported  (Choi et al.,  2008a,b).

The benzofuran unit is essentially planar, with a mean deviation of 0.013 (3) Å from the least-squares plane defined by the nine constituent atoms (Fig. 1). In the crystal structure intermolecualr C–H···O and O–H···O hydrogen bonding interactions are found (Fig. 2 and Table 1). The crystal structure is further stabilized by aromatic π···π interactions between the furan and the benzene rings of adjacent molecules, with a Cg1···Cg2^iii^ distance of 3.841 (5) Å (Cg1 and Cg2 are the centroids of the C1/C2/C7/O1/C8 furan ring and the C2-C7 benzene ring, respectively (Fig. 2).

### Experimental

Ethyl 2-(5-methyl-3-methylsulfinyl-1-benzofuran-2-yl)acetate (303 mg, 1.2 mmol) was added to a solution of potassium hydroxide (337 mg, 6 mmol) in water (15 ml) and methanol (15 ml), and the mixture was refluxed for 5h, then cooled down. Water was added, and the solution was extracted with dichloromethane. The aqueous layer was acidified to pH 1 with concentrated hydrochloric acid and then extracted with chloroform, dried over magnesium sulfate, filtered and concentrated under vacuum. The residue was purified by column chromatography (ethanol) to afford the title compound as a colorless solid [yield 84%, m.p. 461-462 K; *R*_f_ = 0.51 (ethanol)]. Single crystals suitable for X-ray diffraction were prepared by evaporation of a solution of the title compound in acetone at room temperature.

### Refinement

Atom H2 of the hydroxy group was found in a difference Fourier map and refined freely. The other H atoms were positioned with idealized geometry and were refined using a riding model, with C-H = 0.93 Å for aromatic H atoms, 0.97 Å for methylene H atoms and 0.96 Å for methyl H atoms, respectively, and with *U*_iso_(H) = 1.2*U*_eq_(C) for aromatic and methylene H atoms and 1.5*U*_eq_(C) for methyl H atoms.

### Crystal data

**Table:**

C_12_H_12_O_4_S	*D*_x_ = 1.418 Mg m^−^^3^
*M**_r_* = 252.28	Melting point = 461–462 K
Orthorhombic, *P**b**c**a*	Mo *K*α radiation, λ = 0.71073 Å
Hall symbol: -P 2ac 2ab	Cell parameters from 2314 reflections
*a* = 7.767 (1) Å	θ = 2.7–23.2°
*b* = 16.248 (2) Å	µ = 0.27 mm^−^^1^
*c* = 18.733 (2) Å	*T* = 293 K
*V* = 2364.1 (5) Å^3^	Block, colorless
*Z* = 8	0.40 × 0.20 × 0.05 mm
*F*(000) = 1056	

### Data collection

**Table:**

Bruker SMART CCD diffractometer	2690 independent reflections
Radiation source: fine-focus sealed tube	1461 reflections with *I* > 2σ(*I*)
graphite	*R*_int_ = 0.110
Detector resolution: 10.0 pixels mm^-1^	θ_max_ = 27.5°, θ_min_ = 2.2°
φ and ω scans	*h* = −8→10
Absorption correction: multi-scan (*SADABS*; Sheldrick, 2000)	*k* = −20→21
*T*_min_ = 0.899, *T*_max_ = 0.987	*l* = −24→24
13669 measured reflections	

### Refinement

**Table:**

Refinement on *F*^2^	Primary atom site location: structure-invariant direct methods
Least-squares matrix: full	Secondary atom site location: difference Fourier map
*R*[*F*^2^ > 2σ(*F*^2^)] = 0.052	Hydrogen site location: difference Fourier map
*wR*(*F*^2^) = 0.156	H atoms treated by a mixture of independent and constrained refinement
*S* = 1.04	*w* = 1/[σ^2^(*F*_o_^2^) + (0.0469*P*)^2^ + 5.6706*P*] where *P* = (*F*_o_^2^ + 2*F*_c_^2^)/3
2690 reflections	(Δ/σ)_max_ < 0.001
160 parameters	Δρ_max_ = 0.35 e Å^−^^3^
0 restraints	Δρ_min_ = −0.45 e Å^−^^3^

### Special details

**Table:**

Geometry. All esds (except the esd in the dihedral angle between two l.s. planes) are estimated using the full covariance matrix. The cell esds are taken into account individually in the estimation of esds in distances, angles and torsion angles; correlations between esds in cell parameters are only used when they are defined by crystal symmetry. An approximate (isotropic) treatment of cell esds is used for estimating esds involving l.s. planes.
Refinement. Refinement of F^2^ against ALL reflections. The weighted R-factor wR and goodness of fit S are based on F^2^, conventional R-factors R are based on F, with F set to zero for negative F^2^. The threshold expression of F^2^ > 2sigma(F^2^) is used only for calculating R-factors(gt) etc. and is not relevant to the choice of reflections for refinement. R-factors based on F^2^ are statistically about twice as large as those based on F, and R- factors based on ALL data will be even larger.

### Fractional atomic coordinates and isotropic or equivalent isotropic displacement parameters (Å^2^)

**Table:**

	*x*	*y*	*z*	*U*_iso_*/*U*_eq_	
S	0.11771 (12)	0.71553 (6)	0.38153 (5)	0.0201 (2)	
O1	0.2472 (3)	0.49764 (16)	0.45410 (13)	0.0239 (6)	
O2	0.6841 (4)	0.67768 (18)	0.46398 (15)	0.0285 (7)	
H2	0.761 (7)	0.697 (3)	0.437 (3)	0.057 (17)*	
O3	0.5236 (4)	0.6591 (2)	0.36700 (15)	0.0429 (9)	
O4	−0.0703 (3)	0.73974 (16)	0.38751 (15)	0.0304 (7)	
C1	0.1334 (5)	0.6112 (2)	0.40470 (19)	0.0198 (8)	
C2	0.0240 (5)	0.5428 (2)	0.38493 (19)	0.0206 (8)	
C3	−0.1287 (5)	0.5323 (2)	0.34684 (19)	0.0232 (8)	
H3	−0.1837	0.5773	0.3263	0.028*	
C4	−0.1973 (5)	0.4540 (2)	0.3400 (2)	0.0262 (9)	
C5	−0.1128 (5)	0.3867 (2)	0.3718 (2)	0.0263 (9)	
H5	−0.1594	0.3344	0.3664	0.032*	
C6	0.0363 (5)	0.3956 (2)	0.4107 (2)	0.0238 (9)	
H6	0.0910	0.3509	0.4317	0.029*	
C7	0.1005 (5)	0.4747 (2)	0.41678 (19)	0.0201 (8)	
C8	0.2619 (5)	0.5814 (2)	0.44641 (18)	0.0203 (8)	
C9	0.4120 (5)	0.6207 (2)	0.48186 (19)	0.0228 (9)	
H9A	0.4666	0.5806	0.5129	0.027*	
H9B	0.3713	0.6656	0.5115	0.027*	
C10	0.5441 (5)	0.6535 (2)	0.4304 (2)	0.0218 (8)	
C11	−0.3638 (6)	0.4401 (3)	0.2989 (2)	0.0419 (12)	
H11A	−0.4395	0.4860	0.3062	0.063*	
H11B	−0.4183	0.3907	0.3157	0.063*	
H11C	−0.3386	0.4348	0.2490	0.063*	
C12	0.1561 (6)	0.7075 (3)	0.2878 (2)	0.0349 (11)	
H12A	0.0770	0.6687	0.2674	0.052*	
H12B	0.2719	0.6891	0.2798	0.052*	
H12C	0.1400	0.7603	0.2659	0.052*	

### Atomic displacement parameters (Å^2^)

**Table:**

	*U*^11^	*U*^22^	*U*^33^	*U*^12^	*U*^13^	*U*^23^
S	0.0185 (5)	0.0201 (4)	0.0216 (4)	−0.0006 (4)	0.0017 (4)	0.0006 (4)
O1	0.0196 (14)	0.0243 (14)	0.0278 (15)	−0.0018 (11)	−0.0061 (12)	0.0021 (12)
O2	0.0196 (15)	0.0405 (18)	0.0254 (15)	−0.0086 (13)	−0.0019 (13)	0.0023 (13)
O3	0.0280 (18)	0.081 (2)	0.0198 (15)	−0.0110 (17)	−0.0037 (13)	0.0048 (15)
O4	0.0202 (15)	0.0282 (15)	0.0429 (17)	0.0063 (12)	0.0086 (13)	0.0054 (13)
C1	0.020 (2)	0.0197 (19)	0.0195 (18)	−0.0005 (16)	0.0013 (16)	−0.0011 (15)
C2	0.022 (2)	0.024 (2)	0.0159 (18)	0.0014 (16)	0.0032 (16)	−0.0024 (16)
C3	0.023 (2)	0.025 (2)	0.0217 (19)	0.0007 (18)	−0.0037 (17)	0.0020 (16)
C4	0.024 (2)	0.035 (2)	0.0197 (19)	−0.0046 (19)	−0.0036 (17)	−0.0004 (18)
C5	0.033 (2)	0.0207 (19)	0.025 (2)	−0.0072 (19)	0.0007 (19)	−0.0001 (16)
C6	0.024 (2)	0.021 (2)	0.026 (2)	0.0005 (17)	−0.0034 (18)	0.0047 (16)
C7	0.018 (2)	0.025 (2)	0.0181 (18)	0.0005 (17)	−0.0005 (16)	−0.0006 (15)
C8	0.020 (2)	0.025 (2)	0.0169 (18)	−0.0017 (16)	0.0018 (16)	−0.0024 (16)
C9	0.021 (2)	0.028 (2)	0.0186 (18)	−0.0006 (17)	−0.0023 (16)	−0.0018 (16)
C10	0.0144 (19)	0.029 (2)	0.0215 (19)	0.0023 (17)	−0.0009 (16)	−0.0048 (16)
C11	0.037 (3)	0.045 (3)	0.044 (3)	−0.013 (2)	−0.019 (2)	0.010 (2)
C12	0.034 (3)	0.046 (3)	0.025 (2)	0.006 (2)	0.0006 (18)	0.005 (2)

### Geometric parameters (Å, °)

**Table:**

S—O4	1.516 (3)	C4—C11	1.522 (6)
S—C1	1.754 (4)	C5—C6	1.375 (6)
S—C12	1.786 (4)	C5—H5	0.9300
O1—C8	1.374 (4)	C6—C7	1.383 (5)
O1—C7	1.388 (4)	C6—H6	0.9300
O2—C10	1.316 (5)	C8—C9	1.486 (5)
O2—H2	0.85 (5)	C9—C10	1.506 (5)
O3—C10	1.202 (4)	C9—H9A	0.9700
C1—C8	1.356 (5)	C9—H9B	0.9700
C1—C2	1.447 (5)	C11—H11A	0.9600
C2—C7	1.390 (5)	C11—H11B	0.9600
C2—C3	1.394 (5)	C11—H11C	0.9600
C3—C4	1.385 (5)	C12—H12A	0.9600
C3—H3	0.9300	C12—H12B	0.9600
C4—C5	1.408 (5)	C12—H12C	0.9600
			
O4—S—C1	107.41 (17)	O1—C7—C2	110.7 (3)
O4—S—C12	104.62 (19)	C1—C8—O1	110.7 (3)
C1—S—C12	99.26 (19)	C1—C8—C9	133.0 (4)
C8—O1—C7	106.3 (3)	O1—C8—C9	116.3 (3)
C10—O2—H2	114 (3)	C8—C9—C10	113.6 (3)
C8—C1—C2	107.8 (3)	C8—C9—H9A	108.8
C8—C1—S	122.6 (3)	C10—C9—H9A	108.8
C2—C1—S	129.7 (3)	C8—C9—H9B	108.8
C7—C2—C3	119.1 (3)	C10—C9—H9B	108.8
C7—C2—C1	104.5 (3)	H9A—C9—H9B	107.7
C3—C2—C1	136.4 (4)	O3—C10—O2	124.0 (4)
C4—C3—C2	119.1 (4)	O3—C10—C9	124.7 (4)
C4—C3—H3	120.4	O2—C10—C9	111.3 (3)
C2—C3—H3	120.4	C4—C11—H11A	109.5
C3—C4—C5	119.6 (4)	C4—C11—H11B	109.5
C3—C4—C11	120.7 (4)	H11A—C11—H11B	109.5
C5—C4—C11	119.7 (4)	C4—C11—H11C	109.5
C6—C5—C4	122.3 (4)	H11A—C11—H11C	109.5
C6—C5—H5	118.8	H11B—C11—H11C	109.5
C4—C5—H5	118.8	S—C12—H12A	109.5
C5—C6—C7	116.5 (4)	S—C12—H12B	109.5
C5—C6—H6	121.8	H12A—C12—H12B	109.5
C7—C6—H6	121.8	S—C12—H12C	109.5
C6—C7—O1	125.9 (3)	H12A—C12—H12C	109.5
C6—C7—C2	123.3 (4)	H12B—C12—H12C	109.5
			
O4—S—C1—C8	−138.0 (3)	C8—O1—C7—C6	−179.5 (4)
C12—S—C1—C8	113.4 (3)	C8—O1—C7—C2	0.8 (4)
O4—S—C1—C2	42.9 (4)	C3—C2—C7—C6	2.4 (6)
C12—S—C1—C2	−65.7 (4)	C1—C2—C7—C6	−179.6 (3)
C8—C1—C2—C7	−0.9 (4)	C3—C2—C7—O1	−177.9 (3)
S—C1—C2—C7	178.3 (3)	C1—C2—C7—O1	0.1 (4)
C8—C1—C2—C3	176.6 (4)	C2—C1—C8—O1	1.5 (4)
S—C1—C2—C3	−4.2 (7)	S—C1—C8—O1	−177.8 (2)
C7—C2—C3—C4	−1.9 (5)	C2—C1—C8—C9	179.3 (4)
C1—C2—C3—C4	−179.1 (4)	S—C1—C8—C9	0.0 (6)
C2—C3—C4—C5	0.4 (6)	C7—O1—C8—C1	−1.4 (4)
C2—C3—C4—C11	179.9 (4)	C7—O1—C8—C9	−179.6 (3)
C3—C4—C5—C6	0.8 (6)	C1—C8—C9—C10	−66.5 (5)
C11—C4—C5—C6	−178.8 (4)	O1—C8—C9—C10	111.2 (4)
C4—C5—C6—C7	−0.3 (6)	C8—C9—C10—O3	10.1 (6)
C5—C6—C7—O1	179.1 (3)	C8—C9—C10—O2	−171.7 (3)
C5—C6—C7—C2	−1.2 (6)		

### Hydrogen-bond geometry (Å, °)

**Table:**

*D*—H···*A*	*D*—H	H···*A*	*D*···*A*	*D*—H···*A*
C9—H9B···O4^i^	0.97	2.48	3.339 (5)	148
O2—H2···O4^ii^	0.85 (6)	1.74 (6)	2.590 (4)	175 (5)

Symmetry codes: (i) *x*+1/2, −*y*+3/2, −*z*+1; (ii) *x*+1, *y*, *z*.
